# Characteristics of Pressure on the Apophysis in the Course of Paediatric Heel Pain—Preliminary Report

**DOI:** 10.3390/ijerph20075403

**Published:** 2023-04-05

**Authors:** Aleksandra Bitenc-Jasiejko, Anna Walińska, Krzysztof Konior, Kinga Gonta, Piotr Skomro, Konrad Kijak, Małgorzata Kowacka, Danuta Lietz-Kijak

**Affiliations:** 1 Department of Propaedeutics, Physical Diagnostics and Dental Physiotherapy, Pomeranian Medical University, 70-204 Szczecin, Poland; 2 PODOLOGIA.pl, FIKOU Physiotherapy, 44-100 Gliwice, Poland; 3 Doctoral Study Department of Propaedeutic, Physical Diagnostics and Dental Physiotherapy, Pomeranian Medical University, 70-204 Szczecin, Poland; 4 Orthogenic Rehabilitation and Podology Center, 45-573 Wroclaw, Poland; 5 Student Scientific Society at the Department and Clinic of Internal Diseases, Angiology and Physical Medicine, Medical University of Silesia, 40-055 Katowice, Poland; 6 Independent Public Complex of Health Care, 42-300 Żarki, Poland

**Keywords:** Sever’s disease, osteochondritis dissecans (OCD), foot defects, postural disorders, pedobarography

## Abstract

Increased pressure on the heel apophysis is often implicated as a cause of paediatric heel pain. However, there are few reports on the causes of the increased pressure and its origin. Therefore, the aim of this study was to analyse the distribution of pressure on the feet in children with heel pain. The study included 33 paediatric patients with non-traumatic heel pain, i.e., 24 boys (73%) and 9 girls (27%), aged on average 11.2 years (±3 years). Pedobarographic diagnostics proved a decrease in the pressure on the heels in relation to the ground and the transfer of the projection of the centre of gravity to the forefoot. While standing, the average contribution of the pressure on the heel was 0.52, SD = 0.14 in children with normal and reduced weight. In overweight children, the average pressure on the heel was higher (0.60, SD = 0.08), but the small number of children with this characteristic (*n* = 4) did not allow conclusions to be drawn in this area. Heel underload was also demonstrated during gait. However, the assessment of this aspect requires additional observational analyses in the field of propulsion and gait phases. The reduced pressure on the heel promotes apophysis traction, causing intracanal compression. Studies have shown that the causes of apophysis traction may be postural defects (in particular, forward inclination of body posture) and overpronation of the foot, or defects in the metatarsal area.

## 1. Introduction

The calcaneus has two ossification centres, one of which is a primary ossification centre present already at birth. The secondary ossification centre (calcaneal apophysis) appears at 4–7 years of age in girls and at 7–9 years of age in boys [1,2]. Prior to the formation of the apophysis, the appearance of the posterior aspect of the heel bone is serrated. Before fusion, the apophysis has a higher density. The growth plate remains open until at least 14 years of age [3]. The fusion of the calcaneus and apophysis is complete at 15–17 years of age [2,4]. Heel pain can be a symptom of many conditions in the heel region and it is important to differentiate between them, which calls for a precise determination of the causes, symptoms and, consequently, the treatment process [5,6]. The most commonly identified cause of paediatric heel pain is Sever’s disease [7,8]. In 1912, James Warren Sever described the clinical picture, detailing the inflammatory injury to the calcaneal apophysis, which he associated with increased muscle strain in children. Sever also identified changes in radiographic examinations, with an enlargement of the epiphyseal line of the ossification nucleus of the calcaneus [9]. Just a few years after Sever’s report, inflammation was defined as the cause of pain [10,11,12]. Consequently, Sever’s disease is classified as osteochondrosis (OCD) [13]. OCD occurs in children from the age of 8 years, more often in boys (mean age 10 years, in girls 12 years) [14,15,16]. The onset may be as late as 15 years, which is more likely to occur in boys [7,8,17]. Symptoms may occur unilaterally, but bilaterality has been reported in more than 60% of cases [18,19,20]. It typically occurs during growth spurts, particularly during calcaneal ossification, and is much more common in children involved in sports [12,16,18,20,21,22,23]. A growing incidence of calcaneal apophysitis was reported in a year-on-year analysis between 2008 and 2010, which was also significantly higher compared to other studies on heel pain [6,24,25,26].

## 2. Aetiology

The OCD aetiology is not entirely clear [27,28,29]. Osteochondrosis has been characterised as ischaemic necrosis of chondrocytes of hyaline cartilage and its mineralisation (local calcification) [30,31,32]. OCD may result from trauma, overuse or other diseases affecting normal bone growth [33]. Given that sports overuse injuries have been implicated as a predisposing factor for OCD (Sever’s disease), the majority of research studies have focused on this group [12,18,21,34,35,36,37,38]. In sports, the overuse syndrome is mainly attributed to running and plyometric training [39,40,41,42,43]. In this respect, the main factors identified as leading to overuse lesions include training errors (especially a lack of exercise variety), sports shoes (e.g., too tight), hard or unstable training surfaces, etc. [3,28,34,44,45,46,47]. One important aspect of sports training is that children who run intensively develop muscle imbalances, which can lead to excessive local bone loading [48,49].

The calcaneal apophysis is subjected to greater axial loads than the epiphysis [7]. Inside is the growth plate, which grows more slowly than the epiphysis [50,51]. The apophysis serves as the attachment site of the Achilles tendon and the plantar fascia. The ischaemic nature of the abnormalities in the heel area was suggested as early as 1926, on the basis of clinical and radiographic findings. At that time, ischaemia was believed to be caused by inflammation provoked by a mechanical factor, i.e., traction (pulling) of the Achilles tendon and plantar fascia in opposite directions [52,53]. This mechanism exposes the growth plate and the apophysis to high stress, which can lead to aseptic ischaemia [8,27]. The peak incidence of Sever’s disease, i.e., 8–15 years of age [7,8], coincides with the ossification process of the posterior calcaneus [53]. Rapid bone growth in children during this time predisposes them to the development of calcaneal apophysitis [3,54]. The developing skeleton is more susceptible to injury because the bones are much more porous, and their structure around the epiphyseal plate is weakened [55,56,57,58]. Bones also tend to grow faster than muscles and tendons, which can lead to reduced mobility and increased tightness, especially at the attachment sites [49,59,60].

Ischaemia within the calcaneal apophysis may result from individual anatomical features, such as a lack of blood vessels or other circulatory disorders [27,61]. In assessing the genetic basis of OCD, it has been suggested that the lesions may be caused by abnormal chondrocyte matrix synthesis, which may be the cause of abrupt endochondral ossification [31]. Matching lesions have been observed in monozygotic (identical) twins [62]. However, genetic factors are still unexplored [63].

## 3. Diagnostics

A preliminary diagnosis of OCD is based on medical history and a thorough physical examination [16]. Complaints of a ‘dull’ pain appearing in the posterior lower part of the calcaneus [64,65] during weight-bearing or after exercise [28,66] can initially distinguish OCD from plantar fasciitis and Achilles tendonitis. However, it is essential to carry out a palpation examination and a squeeze test in the area of the calcaneal apophysis, which will be positive in apophysitis [16,44,67,68]. Visually, some structures may appear swollen [56,69], which will significantly differ from the swelling in Haglund’s syndrome, at least in terms of location [70,71].

Many authors have stated that Sever’s disease is a clinical diagnosis [64,68] and that X-ray imaging does not provide a basis for the diagnosis. Despite the presence of clinical symptoms, radiological findings may be interpreted as normal [72] and there are many discrepancies in the interpretation of radiological images [48,73]. This may be due to developmental anatomical differences [27,74]. X-ray images should be taken for both feet, even when there are no lesions in both heels, so that individual characteristics can be compared [75]. In the course of OCD, lesions can form underneath healthy cartilage [28,68]. Signs of subchondral bone necrosis with the overlying cartilage still intact have been noted in a number of studies [32,76,77,78,79,80]. X-ray imaging may, however, be important in the differentiation of conditions presenting with heel pain [4,68,81]. These include plantar fasciitis, Achilles tendonitis, supernumerary bones, cysts, bone coalitions, fractures (traumatic or stress-induced), hyperdensity in the course of systemic diseases requiring different treatment, etc. [3,50,81,82,83,84]. Diagnostic decisions are made more difficult in view of the research observation that X-ray images of children with pain do not differ from those of healthy children and that no typical necrotic lesions were found in the apophysis [53]. The same features were shown to be present in X-rays of children with heel pain as in asymptomatic children., i.e., in a group of 148 patients, slots (i.e., apophyseal fragmentation) were observed in 26% of X-rays and hyperdensity in 89% of children [1]. On the other hand, a number of research papers on Sever’s disease reported apophyseal thickening, fragmentation and hyperdensity as important findings [14,18,34,85,86,87,88]. Ultrasound can be used both in the assessment of soft tissue lesions (bursitis, Achilles tendonitis, plantar fasciitis, etc.) and to track treatment progress and observe apophyseal fragmentation [28,73].

The disease is not commonly diagnosed using MRI due to the cost [89]. However, given its ischaemic nature and the need to differentiate between causes of pain, in persistent complaints its use is justified. Among others, the aim will be to detect and differentiate subchondral osteopenia, bone marrow oedema and osteomyelitis, tumours, cysts, etc. [30,90,91,92,93]. MRI can detect articular cartilage thickening, lesions, fibrosis, etc. When evaluating questionable lesions, contrast is administered to facilitate differentiation [63]. Some features of structural changes in Sever’s disease have been demonstrated by histological studies [80,94].

According to the literature, OCD diagnosis relies mainly on a detailed medical history and tests aimed at pain differentiation, while X-ray imaging appears not to confirm the diagnosis conclusively. Despite the long history of Sever’s disease, most publications emphasise its unclear aetiology. The main causes include increased pressure on the calcaneal tuberosity. The authors of this publication observed that in children with a diagnosis of OCD, ground reaction forces are not increased and even the opposite is true, i.e., in pedobarographic examination, plantar pressure (pressure between the foot and the floor) may be reduced. This practical conclusion became the starting point for the authors’ analyses focused on the identification of causes within the human body (individual characteristics).

## 4. Main Issue

### 4.1. Objective

Analysis of foot pressure distribution in people with paediatric heel pain.

### 4.2. Study Group

The study group consisted of 33 paediatric patients with non-traumatic heel pain, including 24 boys (73%) and 9 girls (27%), with a mean age of 11.2 (±3 years). Patients presented to medical facilities in Poland with a diagnosis of OCD (Sever’s disease) in order to start rehabilitation and/or obtain customised pressure-relieving orthotics.

### 4.3. Methods

**Figure A1:**
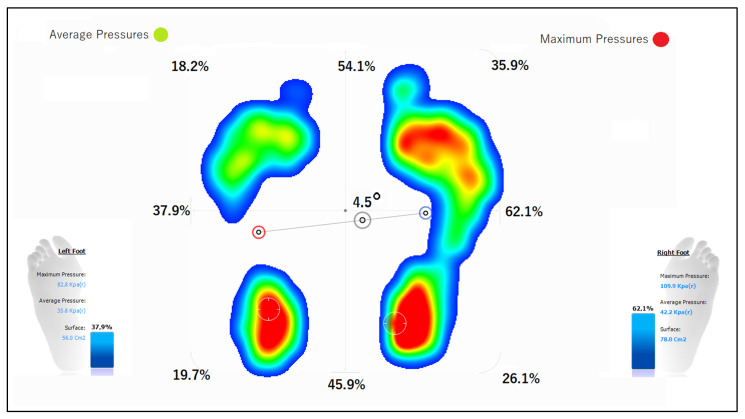
Pedobarography during standing—assessment of centre of gravity (front–back and left–right) in a standing posture (heel pressure = 45.9%, suggesting a forward shift of the centre of gravity).

**Figure A2:**
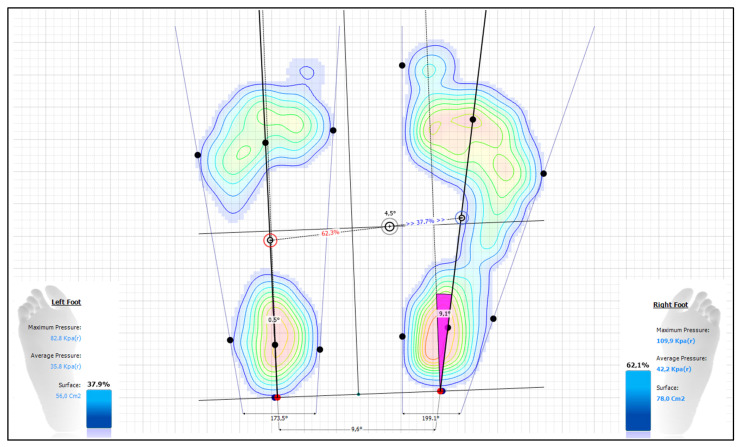
Pedobarography during standing—assessment of foot alignment, angles of abduction (right foot normal abduction, left foot adduction) [121] and above-normal angles of foot proportions (gamma angle—normal range 15–18° [122,123,124]), indicative of the metatarsus adductus (i.e., forefoot adduction).

**Figure A3:**
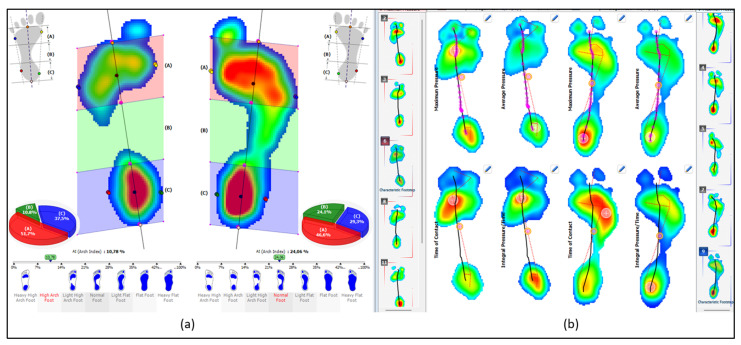
Results of pedobarographic examination of foot arches, the so-called arch index, AI (normal range: 21–28%) [125]: (**a**) measured during standing; (**b**) during walking—finding lack of contact in the lateral foot compartment (i.e., abnormal contact of the lateral compartment with the ground), which confirms the pes planovalgus diagnosis from the visual examination (photogrammetry).

**Figure A4:**
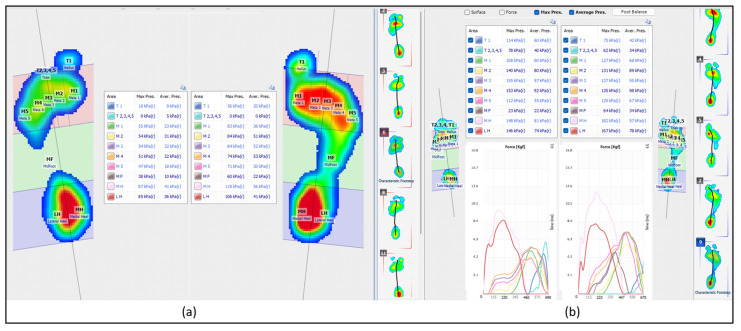
Pedobarographic imaging: (**a**)during standing, (**b**) during walking—repeatable results were used for analysis of hindfoot, midfoot and forefoot: MH—medial hindfoot and LH—lateral hindfoot (normal range: the physiological tarsal valgus in individuals over 8 years of age of 5 degrees translates to increased MH pressure by 15% [126]); the result indicates above-normal pressure in the medial compartment (MH), confirming the finding of tarsal valgus in the course of pes planovalgus. MF—midfoot; reduced pressure, indicative of overpronation (pes planovalgus). M1–5 metatarsophalangeal joint metaplanes; increased pressures are observed at M2–M4 compared to M1 and M5, representing a transverse arch collapse. T1—first toe and T2–5—toes 2–5; result indicates abnormal participation of the toes in the support function.

**Figure A5:**
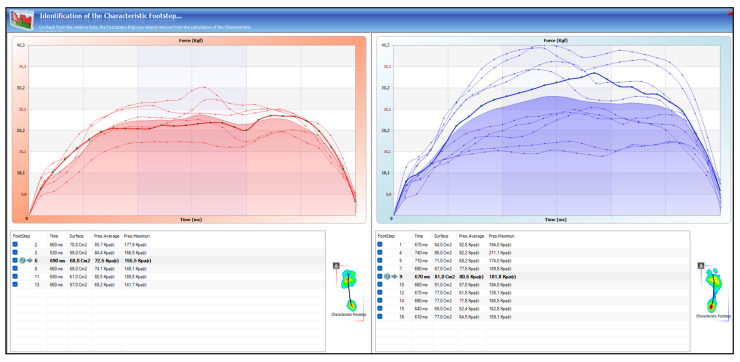
Pedobarographic imaging shows pressure forces during the respective phases of gait; the result indicates abnormal heel strike (delayed landing with midfoot and forefoot involvement [127,128]). The result was confirmed with time-lapse images shown in Figure A6.

**Figure A6:**
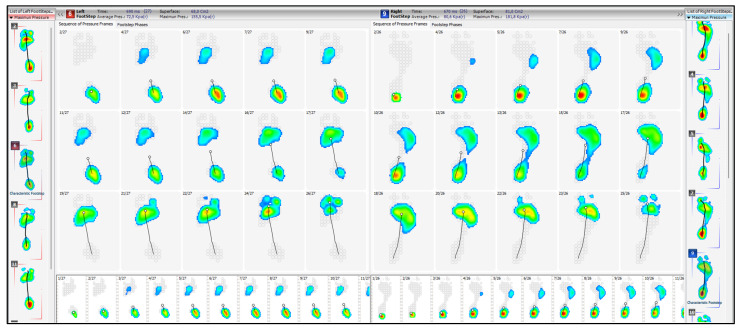
Pedobarographic imaging showing time-lapse images of foot rollover; the result indicates forefoot involvement in stage 1 of foot rollover, without midfoot involvement, which is associated with overpronation characteristic of pes planovalgus.

**Figure A7:**
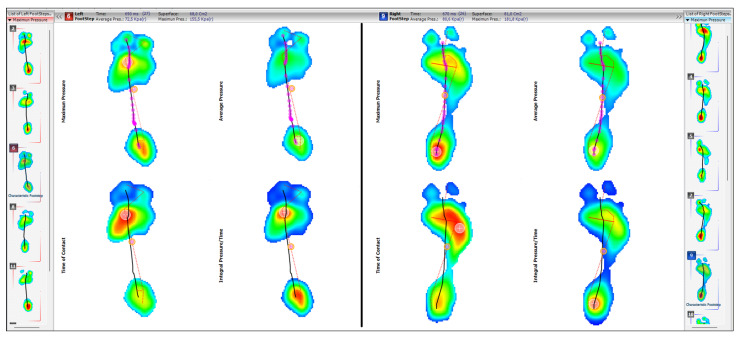
Pedobarographic imaging showing centre of pressure progression (the thinner red dashed line) in four diagnostic aspects: maximum pressure, average pressure, contact time, pressure over time. The result confirms abnormal heel strike (lack of heel eversion), lack of foot supination under full loading (the line runs too medially [129,130]) and significant forefoot overload (especially at MTP 2–3), together with chaotic lines indicating midfoot and forefoot instability.

The study of pressure distribution was carried out using pedobarography (EPS R2 pedobarograph, BIOMECH STUDIO software—sample test results are included in Appendix A: Figure A1, Figure A2, Figure A3, Figure A4, Figure A5, Figure A6 and Figure A7:

1. Assessment of centre of gravity in anteroposterior and lateral view in a standing posture (Figure A1).
2. Assessment of foot alignment—angle of abduction (Figure A2).
3. Assessment of arches using the Arch Index, performed while standing and while walking (Figure A3).
4. Assessment of pressure distribution in individual regions of the foot while standing and while walking (Figure A4).
5. Analysis of all steps (mean pressure forces in individual phases of gait—Figure A5, including time-lapse images—Figure A6).
6. Assessment of centre of pressure progression (gait line) to assess the stages of stance phase (including the involvement of the hindfoot, midfoot and forefoot, quality of the centre of pressure trajectory)—Figure A7.

For inference (i.e., the correctness of differentiation) regarding the relationship between foot pressure distribution disorders and foot and body posture defects, the study was supplemented with physical examination of the feet using goniometry and selected stages of the 6-point FPI (Foot Postural Index) scale [95,96,97,98].

Complex defects that may have an impact on overloading within the calcaneal tubercle were supplemented with the radiological documentation analysis (X-ray):

- Skewfoot or z-foot [99].
- Instability of the 1st radius determined by increasing the angle between the axes of the 1st and 2nd metatarsal bones (IMA—intermetatarsal angle) [100].

In terms of postural physiology, the body axis in the sagittal plane should run vertically from the external auditory meatus, via the acromion, greater trochanter and head of fibula (lateral ankle). The centre of gravity of the human body projects along the front of the lower leg, approximately 4.5 cm from the axis of the ankle joint [101]. The results of the physical examination are summarized in Table 1.

## 5. Statistical Analysis

Mean intragroup differences were assessed using Student’s t-test. The Shapiro–Wilk test was used to check the normality of the data distribution, and Pearson’s coefficient to check correlation between continuous variables. The results were considered statistically significant at *p* < 0.05. The *R* statistical package version 4.1.2 (The R Foundation for Statistical Computing, Wirtschaftsuniversität Wien, Vienna, Austria) was used for the calculations.

## 6. Results

In assessing the distribution of plantar pressure, the distribution of pressures on the feet was measured and analysed in the anteroposterior aspect (front–back, i.e., forefoot vs. hindfoot) and the lateral aspect (left vs. right foot). The results are presented in Table 2 and then, respectively, in Table 3 and Table 4, presenting statistical analysis depending on the participants’ weight. The figures in the tables represent the fraction of plantar pressure in the anteroposterior and lateral aspect, i.e., percentage value divided by 100.

In the study group, the mean forefoot loading expressed as a percentage amounted to 47.0% (standard deviation 14.0%, range (3.9%; 76.0%)). The mean hindfoot loading was 53.0% (standard deviation 14.0%, range (24.0%; 96.1%)). Thus, a clear forward shift of the centre of pressure was observed, which may be related to the forward tilt of the body noted in 75% of the participants (Table 1). This may significantly affect the traction on the calcaneal apophysis through the interplay of the Achilles tendon and plantar structures of the feet. The current analysis of the research results carried out by the authors showed that the average pressure on the feet in the anterior–posterior approach varies significantly between individuals with normal and reduced weight (WEIGHT = N/L) and overweight and obese people (WEIGHT = H). Therefore, separate statistical analyses were performed for both groups, and the results are summarized in Table 3 (WEIGHT = Normal/Low) and Table 4 (Weight = High), respectively.

Statistical analysis of patients with normal (N) and low (L) bodyweight showed reduced pressure in the hindfoot area. This is quite the opposite of the findings in patients with overweight. The small sample of overweight people (*n* = 4) does not allow for statistically significant conclusions, but these findings nevertheless illuminate an important research aspect, related to the potential role of overuse in this condition. These people maintained pressure on the heels, and did not show a forward inclination of the body. Despite a balanced distribution of pressures compared to reference values, high-weight participants showed significant anterior pelvic tilt.

**Figure 1:**
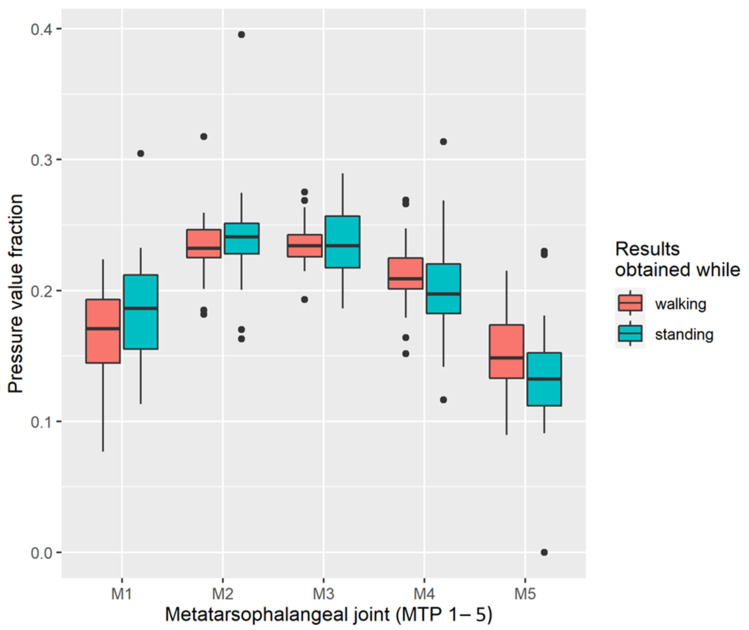
Distribution of maximum pressure on respective metatarsophalangeal joints (fraction index).

The examination of the forefoot was detailed by analysing the meta-planes of individual areas of the metatarsophalangeal joints (MTP I–V), i.e., within the transverse arch. Given that the amount of pressure is dependent on patient weight, in order to determine the statistical distribution of maximum pressure in respective joints across the entire study group, an index was determined, representing the percentage share of each area. The distribution of fractions for each MTP area while standing and walking is presented in Figure 1.

The results of pressure distribution along the transverse arch of the foot show that the second and third metatarsophalangeal joints represent the highest fraction. This confirms the finding of a transverse arch collapse. The largest spread was observed at MTP-1, which may be related to the instability of the first foot radius, found in 78.79% of patients (u1).

The longitudinal arch was also analysed, because the lowering of the plantar of the foot can have a significant impact on the tensile voltages around the attachment structures to the heel apophysis. The results of the pedobarographic examination of the longitudinal arch, determined using the Arch Index (AI) and expressed in %, are shown in Table 5 and Table 6.

The majority of the participants had low AI, i.e., 57.57% in the left foot and 42.42% in the right foot. It should be noted that the AI indicator decreased during walking. It should be noted that the AI indicator decreased during gait, which was related to the fact that most respondents (i.e., 96.87%) showed the features of pes planovalgus in a physical study. During gait, the reduced AI indicator was demonstrated in 63.63% of the left feet and 57.57% right feet.

The hindfoot position was also analysed. The distribution of pressure on the medial (MH) and lateral (LH) heel was investigated using pedobarography. With 5 degrees of eversion, the pressure in the medial heel is 15% higher [98,102,103,104,105,106]. In the study, an analysis was made in the scope of “by how much is MH greater than LH” in the study group (therefore, if MH < LH, the result was negative). The results of the analysis are presented in Table 7.

The results of the MH/LH comparison test show that the pressure while standing in the MH region is higher by on average 11.3% (left foot) and 12.18% (right foot). The spread between the minimum and maximum values is much greater in a standing position. During walking, the pressure in the MH region is on average about 8% higher than in the LH, and the spread between the minimum and maximum values is also smaller compared to the results obtained during standing.

## 7. Discussion

The aetiology of Sever’s disease (OCD) is still unclear, and this state of affairs significantly affects the prevention and treatment of the disease. Starting from Sever himself (1926), it has been repeatedly pointed out that the main cause of the disease is increased pressure on the tissues, but the causes of its origin have not been differentiated. It was also indicated that foot and posture defects may predispose patients to increased pressure and, as a result, damage to the ossifying apophysis of the calcaneus and ischemia [52,53]. However, the correlation between specific foot and posture defects with the type of pressure disorder was not analysed.

To date, research studies on the contribution of ground reaction forces on the heel have produced divergent results, i.e. when investigating the causes, some point to increased pressure on the heel [35,107], while others to the so-called calcaneal atrophy induced by disuse [53]. Becerro de Bengoa Vallejo et al. (2011) indicate that increased pressure on the sole of the feet may be related to Sever’s disease, but it has not been clearly identified whether it is a cause or a symptom of the disease [108]. In research from 2018, the team of Rodríguez Sanz et al. also indicated a slower movement of the body’s centre of gravity in people with Sever’s disease, in addition to intensified pressure [35]. However, neither study indicates the cause of pressure distribution disorders. Contrasting results were shown by Volpon et al. (2002), pointing to the so-called “disuse atrophy”, based on the radiological observation of reduced primary and secondary ossification nuclei and their reduced density [53]. However, this study did not observe the pressure distribution within the calcaneal tubercule.

While standing, the pressure on the heels should be 60% of the total pressure on the feet. The results obtained from our research in this area in the group of 33 patients with heel pain clearly point to reduced heel loading in the majority of the participants, thus shifting the projection of the centre of gravity to the front of the feet. This observation does not apply to patients with an above-normal weight, which requires further analysis, due to the small number of these patients (*n* = 4). Once high-weight patients (weight = H) were excluded from analysis, the mean heel pressures were reduced relative to the forefoot. The average value was 52%. Our physical examination of the posture in the sagittal plane (i.e., the acromion [101]) showed that 75% of the subjects had a forward-leaning posture. Anterior tilted posture is an important determinant of tension, both within the Achilles and plantar aponeurosis.

Shifting the centre of gravity forward involves the posterior band, which causes increased Achilles tendon involvement with an upward force [109,110]. This enhances the traction effect of the calcaneal apophysis, which is extended forward on the sole of the foot and upward on the lower leg. The tractive nature of the tension is not necessarily related to increased pressure on the heel in relation to the ground. However, it may cause increased intratissue pressure. The traction nature of the pressure on the calcaneal tubercle was also indicated by the authors of scientific publications, starting with Lewin’s studies, cited many times (1926). Belikan et al. (2022), in the area of Sever’s disease, defined a syndrome of strain and microtrauma resulting from the traction effect of the Achilles tendon on the apophysis of the calcaneus [37]. However, the authors emphasized that the research failed to determine the causes of this traction. James et al. (2010) indicated that the increased tension in the area of the apophysis of the heel, in the complex of the triceps muscle and the Achilles tendon, may be caused by rapid growth [111].

It should be noted that transferring the projection of the centre of gravity to the forefoot (which takes place, for example, in the take-off phase), causes the plantar flexors that have attachments near the calcaneal tuberosity to undergo eccentric extension, which promotes traction forces within the apophysis [112,113]. Increased pressure within the forefoot was subjected to a detailed analysis, assessing the pressure within the transverse arch and in the distribution in the areas of the metatarsophalangeal joints (MTP I–V, marked as M1–5 in pedobarographic results). The statistical analysis of the research showed significantly greater pressure on the metatarsophalangeal joints M2–M4, compared to MI and MV. This means a significant lowering of the longitudinal arch, and an abnormality in this area may favour the increase in eccentric forces of plantar structures having their initial attachment in the apophysis of the calcaneal tuberosity. The instability of the first ray is one of the causes of Sever’s disease [109,114].

**Figure 2:**
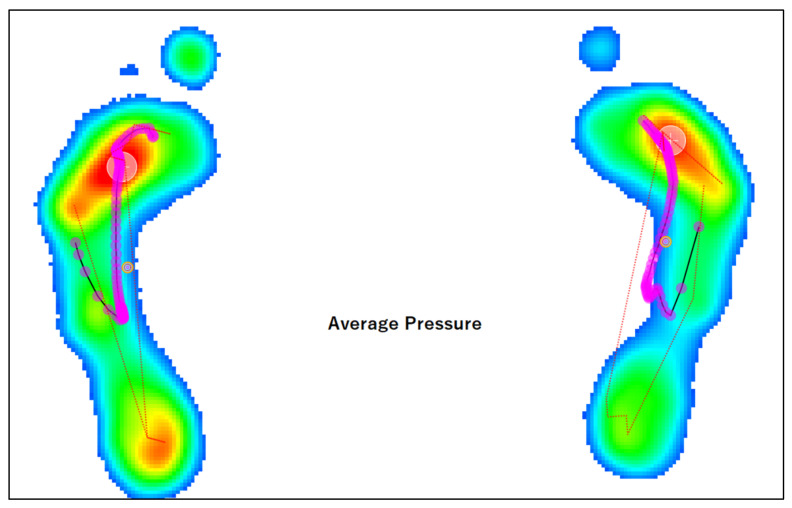
Pedobarographic test result of the propulsion line of the foot, with the return line (landing on the forefoot).

Concluding on the results of heel load pressure during walking in this study required the use of additional observations of pedobarographic results. The distribution of pressure on the medial (MH) and lateral (LH) heel was investigated using pedobarography. 5° of hindfoot eversion corresponds to a 15% higher pressure in the medial part of the heel (MH—medial heel) [98,102,103,104,106]. The results of the MH/LH comparison test show that the pressure while standing in the MH region is higher by on average 11.3% (left foot) and 12.18% (right foot). The spread between the minimum and maximum values is much greater in a standing position. During walking, the pressure in the MH region is on average about 8% higher than in the LH, so the difference between MH and LH during gait decreased. This result was surprising mainly due to the fact that the AI was reduced in most of the subjects (i.e., 57.57% in the left foot and 42.42% in the right foot) and decreased during walking (63.63% in the left foot and 57.57% in the right foot). The result should be interpreted in conjunction with physical examination, mainly because the index is reduced when the lateral compartment of the foot is not in contact with the ground. This may be the case in hollow feet, pes planovalgus and in the hyperpronation of the feet, in the course of internal rotation of the thigh and external rotation of the shank [115]. Upon physical examination, more than 96% of the participants exhibited above-normal parameters of pes planovalgus. Thus, if the subjects had significantly flat valgus (i.e., overpronated feet) and at the same time no results of increased pressure on the medial part of the heel were recorded, the logical conclusion is that there was no full heel landing. This conclusion was confirmed by a detailed analysis of foot rolling phases as a result of a time-lapse pedobarographic examination (example in Figure A6). The above analysis indicates that the research used to record the pressure values must be complemented with a physical examination and observations of the propulsion curve, phases of foot rolling, etc. Detailed analysis of the foot propulsion curve allowed the observation of the return movement of the foot, as shown in Figure 2. Such results occurred unsystematically in the subjects, which made it impossible to draw conclusions in this area. Whether it is a cause or a consequence of paediatric heel pain has not been demonstrated in this study.

However, non-physiological movement towards the toes results in the excessive involvement of plantar structures attaching at the calcaneal tuberosity, and, in addition, the contact time with the ground is significantly prolonged, which further contributes to trauma in these plantar structures [110,116].

As indicated above, a significant number of subjects showed the feature of flat valgus feet. It should be emphasized that foot overpronation is a direct determinant of calcaneal tuber traction [18,29,49,108,117]. It is also indicated as one of the causes of Sever’s disease. This does not exclude the involvement of other defects in the causes of Sever’s disease, because each defect causing increased metatarsal involvement leads to overloading of the forefoot, and these are other aspects that may lead to calcaneal tuber traction [114,116,118]. Studies also indicate the coexistence of Sever’s disease with equinus (Szames et al. (1990)); however, they concern the incidence of Sever’s disease in people with equinus. The need to correct the defect with custom-made foot orthoses during the treatment process of inflammation of the calcaneal apophyse was indicated in the studies by Alfaro-Santafé et al. (2021) and Perhamre et al. (2011). These studies indicate that the relief alone may be insufficient. Although not directly, these studies indicate the role of the defect in the development of OCD.

The analysis of the results showed that both the forward tilt of the posture and the observed defects and dysfunctions of the feet can cause the formation of traction forces within the heel apophysis. Lack of heel load and shifting the body weight forward (during standing and walking) causes tension of the plantar flexors of the feet and eccentric tension within the Achilles tendon [119,120]. Thus, through the action of internal forces, i.e., in the course of the plantar structures of the feet to the front of the foot and upwards in the course of the Achilles tendon, this causes pressure on the heel bone, lifting the heel and stretching in opposite directions. Pedobarographic diagnostics, combined with the assessment of the structure of the feet and posture, allowed for extensive observations, constituting a preliminary report for further research.

## 8. Conclusions

1. In the clinical evaluation of people with paediatric heel pain, it is reasonable to study the distribution of pressures while standing and walking, including conducting a clinical observational evaluation of the detailed results obtained with pedobarography.
2. Reducing the pressure on the heels in relation to the ground illustrates the shift of the centre of gravity to the front. It can also be the effect of bad posture. These factors are an important cause of the formation of traction of the apophysis of the heel and in the relationship of the Achilles–plantar fascia–plantar flexors of the foot, which may result in paediatric heel pain.
3. Traction forces within the heel apophysis cause intracanal pressure on the calcaneal tuber and, at the same time, reduce the contact of the feet with the ground.
4. Pedobarography should be combined with a physical assessment of the feet and body posture for defects that may predispose patients to increased tension of the structures attached to the calcaneal tubercle.

## Figures and Tables

**Table 1:** Results of physical examination (assessment of foot and leg defects), including anthropometric measurements of the feet, selected X-ray parameters of lower limbs (transverse plane) and posture (sagittal plane).

Postural Defect	Side (L-Left/R-Right)	*n*	No Posture Defect	Occurrences of Defects	Type of Postural Defects
Hindfoot	L	32	28 (87.5%)	cv: 4 (12.5%) cvr: 0 (0%)	cv—valgus cvr—varus
	R	32	28 (87.5%)	cv: 5 (15.62%) cvr: 0 (0%)	
Arch defects	L	32	24 (75.0%)	p: 8 (25%) c: 0 (0%)	p—pes planus c—pes cavus
	P	32	23 (71.87%)	p: 9 (28.12%) c: 0 (0%)	
Complex midfoot defects	L	32	1 (3.12%)	pv: 31 (96.87%) e/ev: 0 (0%)	pv—planovalgus e—equinus ev—equinovarus
	R	32	1 (3.12%)	pv: 31 (96.87%) e/ev: 0 (0%)	
	L	32	16 (50.0%)	16 (50%)	metatarsus adductus (forefoot adduction)
	R	32	17 (53.12%)	15 (46.87%)	
	L	32	28 (87.5%)	4 (12.12%)	z-foot (skew foot)
	R	32	28 (87.5%)	4 (12.12%)	
	L	28	2 (7.14%)	26 (78.79%)	1st ray instability
	R	28	2 (7.14%)	26 (78.79%)	
Forward tilt of body		24	6 (25.0%)	fp: 18 (75%)	fp—forward tilt of body

**Table 2:** Results of pedobarographic distributions of plantar pressure—centre of gravity projection.

		Standing Pressure (p)					
Examined Region	Variable	*n*	min	max	mean	st. dev.	95% CI
Centre of gravity projection	Pressure—left	33	0.379	0.757	0.5145	0.0798	(0.4863; 0.5428)
	Pressure—right	33	0.243	0.621	0.4885	0.07658	(0.4613; 0.5156)
	Pressure—front	33	0.039	0.76	0.4695	0.1407	(0.4196; 0.5194)
	Pressure—back	33	0.24	0.961	0.5305	0.1407	(0.4804; 0.5801)

**Table 3:** Analysis of pedobarographic findings of plantar pressure distribution—front–back projection of centre of gravity in patients with normal and low weight.

Variable	*n*	min	max	mean	st. dev.	95% CI
Pressure—front	29	0.039	0.76	0.4792	0.1407	(0.4196; 0.5194)
Pressure—back	29	0.24	0.961	0.5208	0.1407	(0.4804; 0.5801)

**Table 4:** Analysis of pedobarographic findings of plantar pressure distribution—front–back projection of centre of gravity in high-weight patients.

Variable	*n*	max	min	mean	st. dev.	95% CI
Pressure—front	4	0.303	0.478	0.399	0.0854	(0.2633, 0.5352)
Pressure—back	4	0.522	0.697	0.601	0.0854	(0.4648, 0.7367)

**Table 5:** Quantitative results of pedobarographic examination of the arch while standing and while walking (longitudinal arch)—AI.

Arch Index (AI)	*n*	Standing			Walking		
		<21 [%]	21–28 [%]	>28 [%]	<21 [%]	21–28 [%]	>28 [%]
Left foot	33	19	7	7	21	6	6
		AI<% = 57.57%	AI% = 21.21%	AI>% = 21.21%	AI<% = 63.63%	AI% = 18.18%	AI% = 18.18%
Right foot	33	14	8	11	19	4	10
		AI<% = 42.42%	AI% = 24.24%	AI>% = 33.33%	AI<% = 57.57%	AI% = 12.12%	AI>% = 30.30%

**Table 6:** Statistical results of the longitudinal arch of the foot.

		AI While Standing					AI While Walking				
Variable	*n*	min	max [%]	mean [%]	st. dev. [%]	95% CI	min	max [%]	mean [%]	st. dev. [%]	95% CI
Left	33	0	38.63	18.41	10.24	(14.78; 22.04)	0	33.84	16.98	9.987	(13.44; 20.52)
Right	33	0	37.71	21.32	10.33	(17.65; 24.98)	0	44.35	19.71	11.29	(15.71; 23.71)

**Table 7:** Surplus pressure in MH over LH.

	*n*	min (%)	max (%)	mean (%)	SD (%)	95% Conf. Interval
MHoverLH_L standing—left foot	33	−17.24	39.13	11.3	13.03	(6.677, 15.92)
MHoverLH_R standing—right foot	33	−29.63	42.11	12.18	15.46	(6.702, 17.66)
MHoverLH_L_walking—left foot	33	−4.11	30.85	8.006	8.696	(4.923, 11.09)
MHoverLH_R_walking—right foot	33	−5.208	27.27	8.653	8.285	(5.715, 11.59)

Negative figures represent situations where MH < LH.

## Data Availability

The datasets used and/or analysed during the current study available from the corresponding author on reasonable request.
